# Current methods for studying metastatic potential of tumor cells

**DOI:** 10.1186/s12935-022-02801-w

**Published:** 2022-12-09

**Authors:** Pavla Bouchalova, Pavel Bouchal

**Affiliations:** Department of Biochemistry, Faculty of Science, Masaryk University, Kamenice 5, 62500 Brno, Czech Republic

**Keywords:** Migration, Invasiveness, Metastasis, 2D and 3D in vitro assays, In vivo models

## Abstract

Cell migration and invasiveness significantly contribute to desirable physiological processes, such as wound healing or embryogenesis, as well as to serious pathological processes such as the spread of cancer cells to form tumor metastasis. The availability of appropriate methods for studying these processes is essential for understanding the molecular basis of cancer metastasis and for identifying suitable therapeutic targets for anti-metastatic treatment. This review summarizes the current status of these methods: In vitro methods for studying cell migration involve two-dimensional (2D) assays (wound-healing/scratch assay), and methods based on chemotaxis (the Dunn chamber). The analysis of both cell migration and invasiveness in vitro require more complex systems based on the Boyden chamber principle (Transwell migration/invasive test, xCELLigence system), or microfluidic devices with three-dimensional (3D) microscopy visualization. 3D culture techniques are rapidly becoming routine and involve multicellular spheroid invasion assays or array chip-based, spherical approaches, multi-layer/multi-zone culture, or organoid non-spherical models, including multi-organ microfluidic chips. The in vivo methods are mostly based on mice, allowing genetically engineered mice models and transplant models (syngeneic mice, cell line-derived xenografts and patient-derived xenografts including humanized mice models). These methods currently represent a solid basis for the state-of-the art research that is focused on understanding metastatic fundamentals as well as the development of targeted anti-metastatic therapies, and stratified treatment in oncology.

## Introduction

Cell migration and invasiveness play an essential role in a number of biological processes such as embryogenesis, immune response, wound healing, morphogenesis and inflammation [1]. In oncology, these factors are of fundamental importance in the metastasis of tumor cells, which is the most common cause leading to death from cancer. The formation of metastases takes place during a multifactorial and multistage process called metastatic cascade [2, 3]. Its first step is the separation of tumor cells from the primary tumor [2]. Due to the increased expression of proteases, which significantly contribute to the invasive ability of cells by cleavage of the extracellular matrix (ECM), these cells penetrate the basal membrane and invade into the stroma [3]. Stromal cells can increase their aggressive potential and participate in the process of epithelial-mesenchymal transition (EMT) [4, 5], leading to the loss of cellular adhesion, epithelial polarity and an increased migration and invasion capacity of tumor cells. Cells with a mesenchymal phenotype are then able to penetrate the lymphatic or vascular system through a process called intravasation, traveling through the lymph to lymph nodes or circulating in the bloodstream to a pre-metastatic niche in distant organ sites, where they can form a micrometastasis after the extravasation. Micrometastasis can be dormant, or can grow a detectable macrometastasis or secondary tumor [6].

**Fig. 1:**
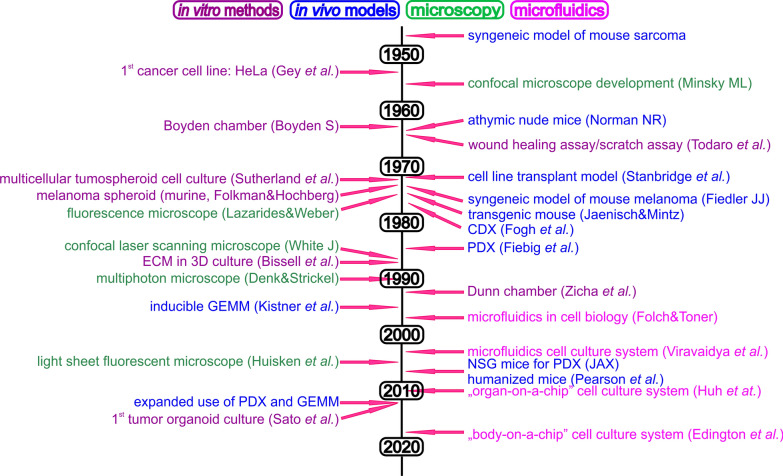
Timeline of biological and technical milestones using in-cell migration and invasion study. CDX cell-line derived xenograft, PDX patient-derived xenograft, NSG NOD SCID gamma mice, ECM extracellular matrix, GEMM genetically engineered mice model

Cell migration is a highly complex process, and a number of biological aspects need to be taken into account when studying it [7]. In vitro model systems based on cell lines are the ones most often used to study migration, however, the conditions in which cells migrate are greatly simplified, and the results may not fully reflect the actual cell behavior in the organism. The study of cell invasiveness is usually performed in a substrate environment that resembles the composition of the natural ECM, such as collagen or Matrigel® (for details, see below). Over the last few years, a great deal of effort has been made to develop in vitro methods that mimic the conditions and interactions present in vivo. In vivo methods in animal models [7] are substantially more experimentally demanding, but are more similar to real conditions in the organism. In order to understand the processes of migration, invasiveness and adhesion in relation to various regulatory mechanisms, new methods are under development. Further, combination with molecular biology, genomic, biochemical and proteomics methods as well as advanced imaging techniques and bioinformatics contribute to this aim. The combination of these approaches forms a toolbox that enables an understanding of complex pathological processes such as the formation of metastases: these methods (Fig. 1) now not only enable the study of the migration of different cell types and their populations, but also the analysis of the role of individual pro-metastatic genes and proteins and their interacting partners and networks. As a result, they can contribute to the development of new diagnostic and therapeutic approaches that will increase the effectiveness of diagnosis, cancer treatment and its personalization. Migration/invasion methods, being artificial systems that are more or less similar to the natural conditions in the organism have their advantages and drawbacks (Table 1), and combining these methods is needed to resolve biological questions.

**Table 1:** The advantages and disadvantages of methods used for migration/invasion study

		Method	Advantages	Disadvantages
2D simple methods (migration)	Lack of tumor microenvironment	Time-lapse microscopy	Very simple method	Only for individual cells; accuracy depends on software analysis
		Wound healing assay (scratch assay)	Easy, quick, cheap, repeatable	Cell stress and retraction of the edges, impact of proliferation, lower reproducibility
		Barrier assay	Easy, quick, cheap, repeatable; decreased cell stress and retraction; higher reproducibility	Impact of proliferation
		Scatter test	Morphological changes and movement in response to HGF	Now not widely used
2D chemotactic method (migration)		Dunn chamber	Directed chemotactic cellular movement	Movement of individual cells is assessed
		Videomicroscopy of cells	Live monitoring of cell movement; assessing of various movement parameters	Instrumentation is needed: camera connected to microscope and incubation chamber
		Chemoattractive beads	Movement of cell sets; comparison of different cell population; ex vivo explant analysis	Now not widely used
2D/3D Boyden chamber (migration/invasion)		Boyden chamber	Cellular phenotype closer to in vivo conditions; active movement of cells through membrane	Process of cell movement is not visualized
		Transwell	Homogenous multiwell platform: different conditions, various cell types or assay settings may be included in one plate; commercially available; high sensitivity to chemoattractant	Limited time of performance
		xCELLigence	On-line monitoring of cells (also including information about proliferation and adhesion); multiwell setting	Special instrument and plates needed
in vitro 3D methods	3D microscopy		Various microscope types according to type of experiment; live imaging—movement of cells in space and time; monitoring of subcellular structures as well as whole organ/organism	Expensive and highly sophisticated instruments; special cultivation plastics; expertized staff
	Microfluidic devices		Movement of cells in the fluid flow mimics body fluidics; customized precisely defined platforms; combination of various environment parameters (cellular, molecular, chemical, biophysical) in one assay; suitable for 3D cell structures	Special instrumentation controlling microflow miniaturized platforms—worse handling; expertized staff
	3D cultures		Cellular phenotype closer to in vivo conditions various type of 3D cell structures; co-cultivation of various cell types (crosstalk); commercial multiwell platform; human tumor cultivation to improve personalized medicine (patient´s consent); state-of-the-art techniques comparable to in vivo mice studies	Long-term manipulation; special cultivation media and plastics; 3D fluorescence microscopy is necessary
in vivo methods	In vivo mice studies		Crosstalk of cancer cell and organism including immune cell in some cases; large offer of various mice strains for various purposes; transplant models of human tumors; therapy testing	Long-term duration of experiments; time-consuming, expensive; special laboratories needed; results not always transferable to human; ethical controversies

## Simple in vitro methods to study cell migration

### Time-lapse microscopy

**Fig. 2:**
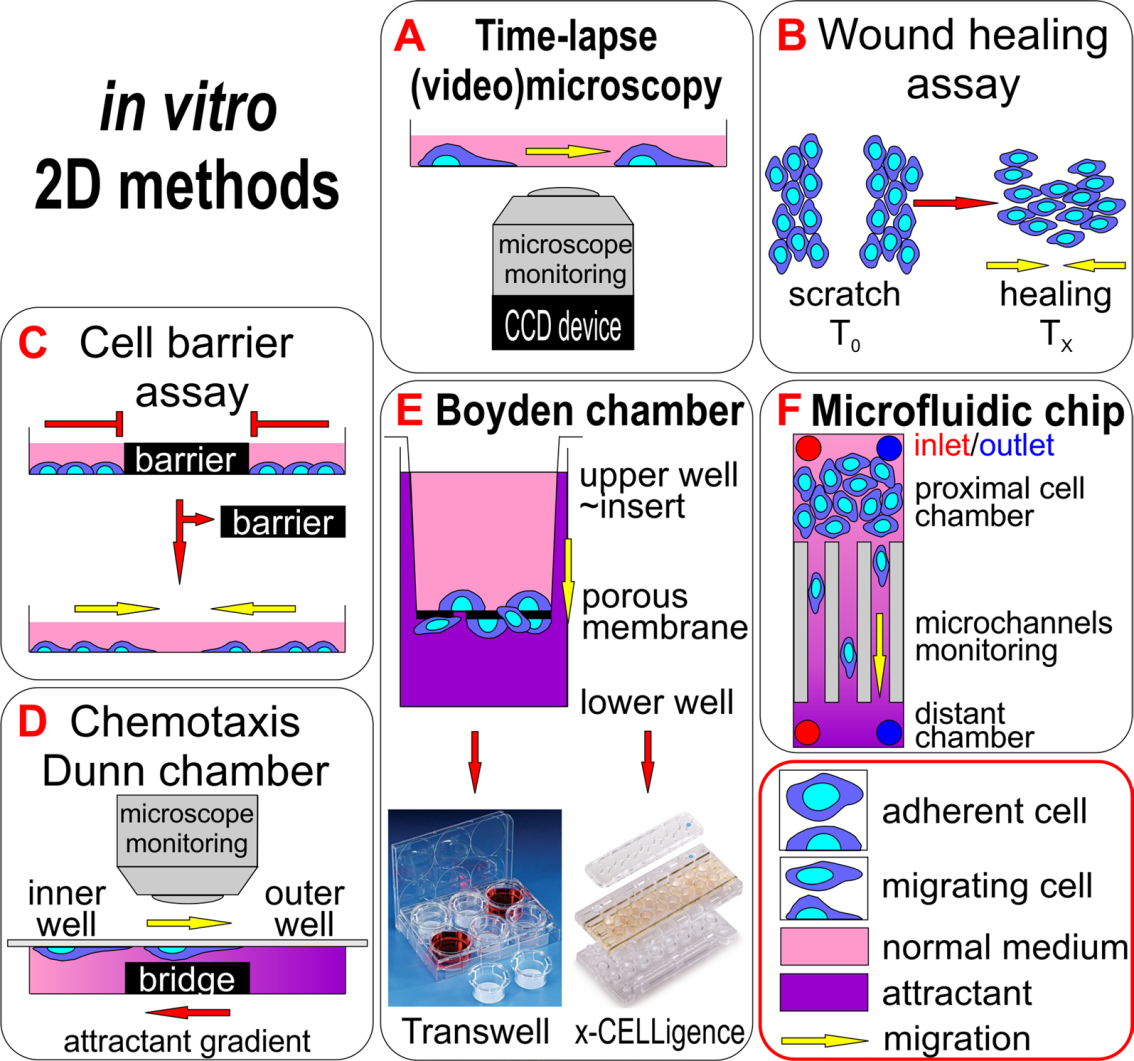
Principles of 2D migration and invasiveness analysis methods according to their simplicity. **A** The easiest method is monitoring cell movement over time using a light microscope or recording cell movement with a camera. **B**, **C** Both wound healing and cell barrier assay methods are based on preparing a defined gap in a cell layer and monitoring the movement of cells to occupy the empty area over time. **D** The Dunn chamber method combines the chemotactic movement of cells and its monitoring over time. **E** The Boyden chamber method is based on cell movement through a porous membrane to a chemoattractant and enables invasion assays in a 3D environment with the usage of ECM components; Transwell and x-CELLigence methods use this basic principle with differences in cell movement monitoring: Transwell with the staining of migrated cells or with migrated cell metabolic activity measurement, x-CELLigence with impedance measurements [186, 187]. **F** Microfluidics provide an additional dimension in migration analysis of the cells by implementing media flow in a defined space (microchannels), mimicking cell behavior in blood or lymph vessels

The easiest way to quantify the migration of individual cells is by monitoring their movement using light microscopy at defined time intervals (Fig. 2A). Photos acquired at different time points are compared to each other using graphical software that enables an overlay of the pictures (e.g. Adobe Photoshop). The length of the migration path is measured and compared between different types of cells or conditions [8].

### Wound-healing/scratch assay

Wound-healing/scratch assay is a commonly used, simple method for measuring the basic parameters of cell migration, such as migration rate and cell polarity [9]. The cells are cultured in a conventional medium until they form a continuous confluent layer. Subsequently, a scratch is created in this layer with a pipette tip of a defined size (Fig. 2B) [10, 11]. Alternatively, a more sophisticated system consisting of a rectangular cultivation dish with a special compatible 36-tooth comb has been recently introduced [12], which results in multiple homogenous and reproducibly prepared scratches in the confluent cell layer. The cells at the edge of the scratch polarize and begin to migrate spontaneously towards the center of the scratch to heal it [13]. With multiple wound edges, they enable the migration of a high proportion of the cells in a population and provide the option of studying biochemical events in migrating cells [12]. The usual way to monitor cell movement in a wound-healing/scratch assay is interval scanning with a conventional optical microscope [9]. Motility can be quantified as the time required to completely heal the scratch, using image analysis software to evaluate the coverage of the scanned area by the cells. The method was originally used for studying the healing process of mechanical wounds. In cancer research, this method is being used, for example, for studying the pro-metastatic genes and proteins that affect the migration ability of tumor cells.

In wound-healing/scratch assay, it is however very important to take into account the contribution of cell proliferation to the healing during the experiment. To deal with this, adding proliferation inhibitors to the medium is an option, e.g. the DNA crosslinker mitomycin C [14], or tracking migrating cells with fluorescent CellTrace labelling (Thermo Fisher Scientific) [15]. The fluorescent dyes bind to the cytosolic proteins, remain in the cytosol and undergo minimal cell-to-cell transfer except for parental-to-daughter cell transfer during cell division. The reduction in signal intensity in daughter cells is constant with each cell division and provides a read out of migrating, non-dividing cells signal normalized to the original cell population signal [15].

The disadvantages of wound-healing/scratch assay mainly originate from stressing the untouched cells with cellular components released from scratched cells, including reactive oxygen species [16–18]. Also, the cells on the border of the scratch may often transiently retract [18], affecting additional biological processes such as anoikis, phagocytosis, membrane repair, and cytokine production, which may increase the level of experimental noise in the assay [16, 19].

### Cell exclusion zone/barrier assay

Some disadvantages of the wound healing/scratch assay mentioned above can be overcome by cell exclusion zone/barrier assay. In contrast to scratch assay, the cell-free area in this method is created with a removable artificial physical barrier in the cell monolayer (Fig. 2C). Four basic types of barriers can be used: a solid removable barrier, degradable gels, a magnetically attachable barrier, and aqueous two-phase systems [20]. The method is more reproducible, thanks to the better defined shape and other parameters of the barrier, which produce delineated cell borders after barrier removal [18, 19]. Multiple barriers can be introduced into one cell monolayer to perform several tests in parallel [18].

### Scatter test

The scatter test is based on monitoring the response of cell lines to hepatocyte growth factor (HGF) [21, 22]. After HGF stimulation, the cells undergo so-called cellular scattering which means scattering from the cell colony as a result of reorganization of the actin cytoskeleton, disruption of intercellular junctions, loss of adhesion to the substrate and subsequent increased ability to migrate. It is a morphological change similar to the EMT process. The progress of the scatter test is monitored with an interval-scanning microscope. The percentage of incoherent/released cells that have undergone cell scattering compared to the total number of cells is evaluated. In this way, the effect of individual gene expression on migration changes can be tested, for example. The method was originally developed using an MDCK cell line derived from normal canine kidneys, but was used to study the role of selected proteins in the EMT in a DU145 human prostate tumor cell line later on [21].

## In vitro methods to study cell migration based on chemotaxis

Chemotaxis is directional cell movement in an extracellular chemical gradient of various compounds, and is of great importance in many biological processes, including the formation of metastases [23]. Chemotaxis can have an effect in a positive manner, when cells migrate towards the high concentration of the chemoattractant (e.g. hormones, growth factors, nutritive supplements), or in a negative manner (chemorepellent), when cells move towards a lower concentration of the compound (e.g. toxins) [24].

### Dunn chamber

The Dunn chamber is a tool that allows the microscopic observation of cell migration in response to the presence of a chemoattractant in real time [25]. The chamber consists of a glass microscope slide with two concentric annular wells. The annular platform that separates the wells (the bridge) is about 1 mm wide, lies exactly 20 μm below the slide's face, and allows microscopic observation of the migrating cells. Thus, when the well is covered with a coverslip carrying the cells to be studied, there is a 20 μm gap between the coverslip and bridge. The inner well of the chamber is filled with control medium, while the outer well is filled with a medium containing a chemoattractant. A radially directed linear diffusion gradient becomes quickly established in this gap, and is subsequently maintained for several hours [26]. The Dunn chamber uses the directed movement of cells from the inner to outer well along the bridge, and this movement is scanned under a microscope and recorded at regular intervals (Fig. 2D). The method is a useful tool for the study of chemotaxis [27], a pro-migration mechanism in cells that contributes to metastasis.

Improved chemotaxis chambers include other type of slides (Insall [28]; Zigmond [29]) that enable direct visualization of the migrating cells. The principle of the chemotaxis gradient in these chambers is the same, but their design is different—Zigmond´s slide has two narrow reservoirs separated by the bridge, while Insall´s slide has two bridges, one 0.5 mm wide and one 1 mm wide, between an inner square reservoir with the control medium and an outer U-shape reservoir filled with the medium containing the chemoattractant [28].

### Videomicroscopy of cells

Videomicroscopy allows the continuous visualization and quantification of chemotaxis, especially in adherent mammalian cells, such as tumor or endothelial cells [30]. This method uses the videomicroscopic real-time or time-lapsed recording of cell movement and software analysis (e.g. Chemotaxis and Migration Tool; [31]) to derive the trajectory and to quantify cell velocity (μm.s^−1^), translocation (μm) and other parameters of the movement (movement direction, pauses during motion) of each individual cell [32, 33], which migrates in a chemotactic gradient usually for up to 24 h. A controlled microenvironment is created thanks to a system of channels and chambers under a special microslip (µ-Slide Chemotaxis, e.g. IBIDI; [34]), under which the cells move. This method can be used, for example, to evaluate the effect of inhibitors on chemotaxis, cell-to-cell chemotaxis, and to distinguish chemotaxis from chemokinesis (random cell movement in a chemical gradient) in cancer cells. Chemotaxis inhibitors are being considered as potential anti-metastatic therapeutics [30].

### Chemoattractive beads

To study the migration of cell sets and for ex vivo cell/tumor explants (living tissue for tissue culture), a method using carriers with chemoattractants was developed. Beads or broken parts of them soaked with chemoattractant molecules are placed on a small dish covered with proteins representing the ECM (e.g. fibronectin) and embedded in nutrient medium, in the vicinity of which the cells or cell explants are subsequently applied. A chemotactic gradient which can induce cell migration is immediately formed around the chemoattractive bead. The cell motility is scanned with a microscope at regular intervals. The method enables the observation of populations of the same cell types as well as comparison of the different chemotactic behavior of two or more labelled cell types at the same time.

### Surface coating materials to improve cell adhesion

The wellbeing of adherent cells in the culture is influenced by their attachment to a substrate. As previously shown, appropriate adhesion (contact of cells with attachment factors) supports cell proliferation, survival and migration properties [35]. Natural coating materials (collagen I, II or IV, laminin, fibronectin, vitronectin) or their artificial alternatives (poly-L/D-lysine, poly-L-ornithine, biocompatible silicone CytoSoft®) can be applied to modify the surface of culture dishes in the majority of methods used to study cell migration to enhance the adhesion of the cells if needed [36].

## In vitro methods to study cell migration and invasiveness based on Boyden chamber

### Cell migration in Boyden chamber

The Boyden chamber was originally developed to study leukocyte chemotaxis, and has become a suitable tool for observing the motility and invasiveness of tumor cells [27]. The classical Boyden chamber consists of two reservoirs separated by a porous membrane (Fig. 2E) [37]. The pores are typically 3–12 μm in diameter, since 8 μm is the optimal size for almost all cancer cells except for lymphocytes [38]. In the upper cylindrical insert, the cells are in a nutrient medium and in the lower well of the culture plate in a medium containing the chemoattractant. The membrane between them is a physical barrier that cells can only overcome through pores by active movement. Non-migrating cells are removed, migrating ones are stained for their quantification.

### Matrices mimicking ECM environment to study cell invasiveness

To study cell invasiveness, the application of matrices mimicking the natural composition of the ECM is essential to preparing a suitable 3D hydrogel environment that supports appropriate cell growth, invasion and the formation of multicellular structures. Generally, the ECM consists of fibrous proteins (e.g. collagens, laminin, fibronectin, elastin, tenascin) and branched proteoglycans (e.g. decorin, syndecan, perlecan) [39]. Fibrous proteins themselves or commercially available gelatin mixtures such as MaxGel™ (Merck), GelTrex (Thermo Fisher Scientific) or Matrigel® (Corning Life Sciences) have been used to form the 3D invasion environment. The widely used Matrigel® is a trademark for a gelatinous protein mixture secreted by Engelbreth-Holm-Swarm mouse sarcoma cells (Corning Life Sciences). Laminin, nidogen, collagen and heparan sulfate proteoglycans with or without growth factors (GF) such as transforming GF beta or epidermal GF [40] are its main components. A disadvantage of using Matrigel® is its slight inconsistency in protein composition, mainly of low abundant proteins, which vary from lot to lot [40]. Non-animal 3D AlgiMatrix sponge made from brown seaweed alginate (polysaccharide hydrogel) is another solution available for this purpose [41].

### Cell invasion using Boyden chamber

Further, the Boyden chamber can be modified to study the invasive properties of tumor cells by covering the microporous membrane with a layer which composition is close to the ECM to form a three-dimensional (3D) environment. Matrices such as collagen or Matrigel® have been used to cover the membrane, where they simulate the ECM during the invasion process. The tumor cells can interact with ECM components and can adhere or proteolytically degrade the ECM substrate. Another advantage is that the cells in the 3D environment are more naturally polar, and their phenotype is therefore closer to in vivo conditions.

### Transwell migration/invasiveness test

Commonly used commercial quantitative in vitro migration and invasiveness tests are largely based on the original Boyden chamber system [42]. Transwell® Invasion Assay is based on disposable plastic multi-well plates with a microporous membrane [43] (Fig. 2E). The porous membrane is manufactured from various materials including polycarbonate, polyethylene terephthalate (PET) and polytetrafluoroethylene, which differ in the size and density of pores, cell attachment properties and suitability for microscopy [44]. As in the original design, the pores in the membrane can be blocked with ECM to study cell invasiveness, which is conditioned by the proteolytic degradation of ECM components. After a selected time period, the cells are visualized by fluorescent staining or fixated and stained with crystal violet. The moving cells count is assessed based on the proportion of cells that have passed through the membrane toward the chemoattractant. The advantage of this method is its relatively high sensitivity—a very low concentration of chemoattractant can induce migration through the membrane, but concurrently, the duration of the studies is limited due to relatively quick balancing of the concentrations between the compartments [45] and the impossibility of visualizing the cells as they move.

### xCELLigence system—a real-time analysis of migration and invasiveness

The xCELLigence system is a well-established technology based on the principle of the Boyden chamber that allows the real-time monitoring of cell motility. The system consists of a Real-time Cell Analyzer (RTCA) instrument including analytical and control units and special disposable cultivation plates (Fig. 2E) that are connected to an electric circuit (Agilent, Santa Clara, CA, USA). Microelectrodes with 22 mV electric potential in electric plates cover about 80% of the surface area at the bottom of the well and form an interdigitating array to provide continuous monitoring of cells. The seeding of the cells into the wells leads to increasing impedance of electric flow, as the cells adhere to the microelectrode surface and obstruct the electric flow. The impedance is a complex quantity describing the component’s apparent resistance to alternating current, and its magnitude is dependent on the number of cells, their size, shape, and cell-substrate attachment quality. These enable continuous monitoring of cell migration, invasiveness, proliferation, cell adhesion and the effect of chemotaxis and cytotoxicity in real time [46, 47]. No staining of the cells at the end of the experiment is needed. Neither the applied electric potential nor the gold microelectrodes affect the viability and adhesion of the monitored cells [48]. “E-plate 16” is a 16-well xCELLigence carrier with simple wells for determining cell adhesion and proliferation. Each well in the cell invasion and migration plate (“CIM-plate 16”) consists of two parts separated by a PET membrane with an 8 μm median pore size and ECM, respectively, with microelectrodes placed upside down directly on the microporous membrane [49]. The output of the experiment performed in the xCELLigence system is the so-called “cell index” (CI): it is a relative quantity, calculated using the following formula: CI = (Zi − Z0)/15 Ω, where Zi is the impedance at a given time point of the experiment, while Z0 is the background impedance of the medium before adding the cells [50]. The CI values for the various parallel experiments are then related to the specific cell property being studied.

## 3D microscopy for visualization of 3D biological objects

To see details and obtain high quality images of 3D biological structures, various designs of microscope mainly based on Z-stack scanning of the object have been developed over the last few decades [51]. The following microscopy techniques are the most commonly used in biological studies. Only their basic principles are presented, as they have various technical modalities and methodological approaches with benefits and limitations for observing biological objects. These techniques are often combined to achieve the best resolution of the image.

### Confocal microscopy

The implementation of confocal microscopy into routine practice in recent years has made it possible to study various cellular processes and sub-cellular structures in detail and enabled the movement of cells in space and time to be recorded [52]. Two basic types of confocal microscopes have been used in biological research: i) confocal laser-scanning microscope (CLSM), or ii) spinning-disc confocal microscope (SDCM) [53]. In principle, the confocal microscope uses fluorescence optics, and its high-energy excitation laser light beam is focused onto a defined spot at a specific sample depth. Pinhole barriers inside the optical pathway cut off the out-of-focus signals, which significantly reduces the noise and provides a bright image. To acquire a plane image of the entire specimen in the CLMS, scanning in a raster is performed. For the SDCM, a rotating disc drilled with multiple pinholes provides multiple excitation beams to be focused on the specimen and emission beams to be detected by a fast scientific complementary metal oxide semiconductor (sCMOS) camera to see the entire field of view at once [53]. CLMS is better for acquiring high-quality and detailed images of subcellular structures (e.g. invadopodia), while SDCM is more suitable for monitoring dynamically changing biological processes in live cell imaging (e.g. movement, migration, invasion) [53]. 3D visualization of the object is achieved by stacking several plane images in suitable microscopy deconvolution software [54]. The resulting fluorescence images can be quantified using specialized software (e.g. ImageJ/Fiji at https://fiji.sc/; [55]). To acquire images in good resolution, special cultivation plastics with 0.17 mm thick glass inserts need to be used instead of plastic bottoms or growing cells directly on the coverslips. Confocal microscopy can be used with all techniques for studying cell migration and invasiveness. With the Boyden chamber invasion setup, the chemotactic invasion of tumor cells is performed, 3D ECM gel with cells is excised, and the rate of migration and invasiveness is then monitored via 3D projection with a confocal microscope and recorded in real time [52].

### Multiphoton microscopy

The most commonly used version of multiphoton microscopy is two-photon microscopy [56]. As confocal microscopy uses a high-energy laser beam, two-photon microscopy uses low-energy near-infrared light to excite the specimen. The fluorophores in the focused spot simultaneously absorb two or more photons with low energy and emit one with higher energy, typically in the visible spectrum, which is detected and multiplied [57]. The use of infrared light and restriction of the excitation to a tiny focal locus result in a high rejection of out-of-focus objects (lower scattering) and deeper penetration down to 1 mm into the object without the use of a pinhole [58–60]. The incorporation of two Titanium:Sapphire lasers of different wavelengths or splitting of the laser beam into two enables simultaneous excitation from blue to far red fluorescent proteins [56]. As excitation is done with low-energy photons, biological objects are subject to lower phototoxicity, including laser-induced damage and lower photobleaching of the fluorophores, which is why this technique is appropriate for live cell imaging and in vivo microscopy over time-lapsed, multiday schedules [56]. Special photoconvertible fluorescent proteins such as Dendra2 change their emitted light after irradiation and enable the tracking of a moving cell within a mouse for a few days [56], this strategy was used for monitoring migrating cell populations expressing different combinations of driver mutations in colon carcinoma [61] and to track single circulating tumor cells [62].

### Light sheet fluorescent microscopy (LSFM)

In this type of microscopy, the expanded laser beam passing through a cylindrical lens is focused onto a thin light sheet perpendicular to the direction of observation [63, 64]. The resolution is intermediate to high, and subcellular structures can be monitored [64]. As only a thin plane of fluorophores is excited in the sample, LSFM setups have the ability to capture dynamic events in living biological samples over prolonged periods of time with less photodamage or fluorophore bleaching, and are significantly faster than standard confocal imaging approaches [63]. LSFM enables the scanning of cultured cells growing on glass or a Petri dish as well as—thanks to long optical pathways—ex vivo whole organs or sedated living model organisms (with or without clearing to increase the fluorescent signal of cancer cells, [65, 66]), each mounted on supports rotatable around the Z-axis in the sample chamber. The environment in the sample chamber can be modified according to the requirements of the experiment or biological object (e.g. cultivation media, immersion media, microfluidics [67]). LSFM has been used in clinical diagnostics of melanoma to reveal metastases in lymph nodes ex vivo [68], for the detection of micrometastasis in murine brains [69] or determining the efficiency of treating lung micrometastases with a conjugate antibody in mice [65].

### ECM degradation assay

The invasion of tumor cells into the surrounding ECM is one of the key steps in the metastatic cascade. ECM degradation assay was developed to determine the local invasion of tumor cells [70]. In this method, artificial gels representing the ECM are labelled with a fluorescent dye. The tumor cells are seeded on the prepared gel, and the formation of cellular invadopodia is observed concurrently with the fluorescent signal loss in areas of degradation and invasion. The tumor cells can be fluorescently stained for the protein of interest to detect invadopodia at invasion sites or to detect the whole cell population [71–73].

## Microfluidic devices

Microfluidics is a fluid-controlling technique with microliter flow in a well-defined design of microchannels and/or microchambers. These devices enable the formation of a 3D environment in which the local cellular (types, numbers, structures and combinations), molecular (adhesion molecules), chemical (material gradients) and biophysical (fluid flow patterns, microenvironment character) parameters can be varied in a controlled manner, both individually and in precise combinations, while analyzing how they contribute to tumor formation, progression, and response to therapy [74, 75]. The devices are highly suitable for the microscopic evaluation of the studied effect.

### Studying cell migration in microchannels

Studying cell migration in precisely defined microchannels of various dimensions, with or without inserted constrictions [76, 77] is a universal and simple method with the option of coating the microchannel surface with various substrates that affect the adhesivity of the migrating cells [33]. The sets of parallel channels are usually custom-made for specific experiments (Fig. 2F), mostly from transparent polydimethylsiloxane (PDMS, dimethicon silicone) [33] known as silicone rubber, or collagen hydrogel [78]. This test is considered to be a 3D method of studying cell migration, as the channel design mimics the natural confinement of the tumor cell in tissue as well as the matrix stiffness [78]. The parameters of the channel, mainly the combination of the height (corresponding also to stiffness) and width, determines the speed of the cell movement, which can be influenced by the applied adhesion molecules, and strongly affects cell shape in the channel [78]. The length of microchannels is variable and also affects cell migration properties. The cells pass through parallel microchannels, and the cell movement is monitored using a light microscope. In this way it is possible to compare the migration of various cell lines, or isogenic cell lines expressing the studied protein with migration potential. In general, it is possible to compare and evaluate different parameters affecting migration in parallel microchannels depending on the design of the experiment.

### Co-culture migration test

Co-culture migration test combines the cultivation of tumor and normal stromal cells such as fibroblasts, epithelial cells, or macrophages in a microfluidic co-culture device to simulate the environment in a real tumor, where various types of cells are combined and influence each other via both chemical signals and direct contacts. The device consists of two or more reservoirs for cell seeding separated by a microchannel array. Various combinations of cell types with defined proportions of tumor and normal cells can be seeded into reservoirs, simulating the changing conditions during tumor development according to the experimental design. The migration of cells is monitored in microchannels and quantified. This test is useful for anti-cancer drug screening [79, 80].

### Microfluidics-based extravasation assays

Microfluidics-based extravasation assays were developed to study the extravasation of tumor cells in a perfusing microvascular network. First, a microfluidic device with three independent hydrogel regions separated by media channels is constructed of ECM hydrogel. The generation of a microvascular network in the hydrogel is performed using normal endothelial cells (HUVEC) and normal fibroblasts (e.g. NHLF) supplemented with fibrinogen and thrombin. After the formation of the network, tumor cells are added and their transmigration is monitored in real time by confocal microscopy [81]. Similarly, tumor cell extravasation through the monolayer of HUVEC cells into the ECM hydrogel can be monitored in a microfluidic device [81].

## 3D cell culture in analysis of cell migration and invasiveness

**Fig. 3.**
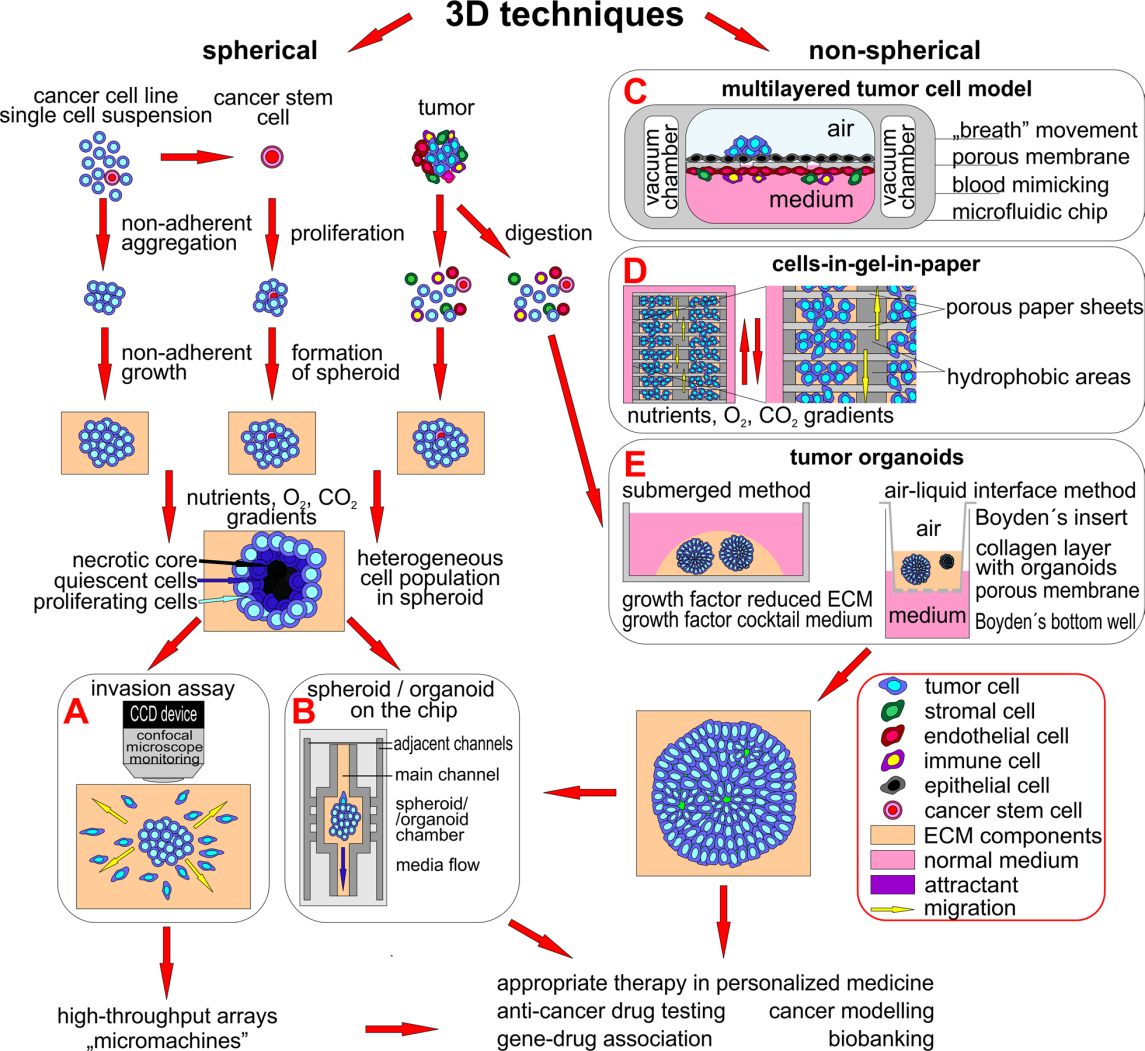
3D methods principles overview. 3D methods can be divided into spherical and non-spherical types based on the shape of the formed cellular structure. Various types of cells can be combined in almost all 3D techniques to better model the natural situation in a human organism. Spherical methods (on the left) use ECM mimicking mixtures to grow spheroids originating from different entities under non-adherent conditions. The mature spheroids can be used in **A** invasion assays, where tumor cells are monitored as they invade into an artificial ECM or in **B** chip methods, where spheroids are placed into a microfluidic device and the influence of media flow or interaction with other cell types (e.g. endothelial cells) through adjacent side channels are monitored. Non-spherical methods (on the right) group layered cell assays and organoids and combine environments for cell structure cultivation. **C** A multilayered tumor cell model is typically used for modeling lung tumor metastasis in vitro: tumor cells can intravasate into the “blood stream” and move to “distant sites”. **D** Cells-in-Gel-in-Paper mimicks multilayered structures with its natural gradients, which influence the phenotype of cells and their movement toward ideal conditions. **E** The state-of-the-art method is the production of tumor organoids grown in ECM components, as they partly maintain the structure and functions of the original tissue and have many research applications. The combination of organoids representing various organs in chambers on one microfluidic chip models tumor cells metastasizing to distant organs

As cells normally grow in space surrounded by ECM and other cells of different types, this method more or less effectively mimics the in vivo natural environment with its biomechanical and physical effects, enables homotypic (one cell type) or heterotypic (more than one cell type; e.g. tumor cells with stromal fibroblast, immune system cells or endothelial cells [82, 83]) interactions between components, and thus provides improved conditions for studying solid tumor cell behavior, including migration and invasion. 3D culturing provides cells with morphology and polarity similar to their in vivo tissue counterparts [83]. 3D models also provide precise experimental entities for studying the effects of anti-cancer drug therapy and further improving personalized medicine. The methods can be divided into spherical (well-rounded morphology of the 3D model) and non-spherical ones (Fig. 3).

### Spherical 3D tumor structures

Spherical 3D cell cultures are the most exploited in vitro model in cancer research [84]. Different biological spherical 3D models in tumor biology research are categorized as (Fig. 3 left part) [83, 85]: i) multicellular tumor spheroids, generated under non-adherent conditions from a single-cell suspension of established cancer cell lines [86], ii) tumorospheres, obtained from an expanding cancer stem cell isolated from a cancer cell line as well as from a tumor [87], iii) tissue-derived tumor spheres, formed of only cancer cells partially dissociated from tumor tissue [88], and iv) patient-derived organotypic multicellular spheroids, grown from finely cut tumor tissue under non-adherent conditions [86].

#### Multicellular tumor spheroids

Multicellular tumor spheroids are based on the tendency of adherent cells to attach to each other and aggregate into multicellular spheroids in a low-attachment environment. Many different methods are used to grow tumor spheroids. The spheroids in a suspension emerge due to i) the agitation of a cell suspension in a spinner flask [83]; ii) the low adhesion of cells to attachment-resistant culture plastic surfaces that induce cell aggregation (e.g. agar [89], agarose [90], polyHEMA [91] or specially prepared polystyrene, which is hydrophilic and neutrally charged, and is now being widely used [92]); iii) the hanging-drop approach [93], or iv) the encapsulation of cells in hollow microcapsules with alginate-based membranes [94, 95]. Hanging-drop spheroids are prepared by pipetting a 20–30 μl drop of cell suspension onto the lid of the cultivation dish, which is then flipped over onto the dish containing the cultivation medium. Gravity causes the cells to form aggregates, which are later transferred to the collagen matrix to grow spheroids [93]. The use of spheroid multi-arrays is possible due to commercially available purpose-built plastic multiwell plates, which support the growth of uniform spheroids and enable high-throughput anti-tumor drug screening [83]. The formation of the spheroid creates a heterogenous population of cells thanks to the natural gradient of oxygen, nutrients, metabolites, or signaling molecules from the surface to the core of the spheroid [96]. Spheroids that are more than 500 μm in diameter start to have necrotic cores due to the above gradients, which is surrounded by a rim of quiescent cells and proliferating cells on the surface of the spheroid [83].

#### Spheroid invasion assay

The principle of this assay is the time-lapsed observation of the spread of tumor cells to the ECM around the parental tumor spheroid. It is useful for the phenotypic comparison of syngeneic cancer cell lines with changed gene expression. The co-cultivation of tumor and normal stromal cells as in a natural tumor microenvironment (TME) provides heterologous interactions in the study, positively influences the invasive capacity of tumor cells, and natural spread of the tumor cells from the spheroid is observed (Fig. 3A) [97].

#### Spheroid-on-the chip

The growth of the spheroids in the microfluidic device in the ECM matrix with continual controlled flow like that of interstitial fluid in the tissue adds another level of control into the 3D system that corresponds to the in vivo mice model. The assay is performed in a reusable glass microfluidic device with a spheroid placed in the microwell to study early cancer spread under the continual perfusion of culture medium, and the influence of the metastatic biomarkers on the metastatic process (Fig. 3B) [98].

#### Spheroid array chip—“micromachines”

This approach is based on the usage of a micropillar/microwell chip with 532 pillars that carry the alginate-embedded spheroids, which grow submerged into the media in microwells [99]. The micropillar chip can be easily replaced into a new microwell chip containing the drug to be tested. The advantages of this method are the high-throughput screening of drug effects, including the inhibition of cell spread to the surroundings, the usage of only 100 cells per spheroid, and micro- and nano-volumes of solutions used. On the other hand, the miniaturization in this approach is highly dependent on the instruments, from the preparation of the chip and spheroids to the automated reading of the results.

### Non-spherical 3D structures

Non-spherical 3D cultures include multilayered tumor cell cultures, tumor slices, organoids and 3D cultures within a reconstituted basement membrane [83].

#### Multilayered tumor cell model (MTCM)

A MTCM is typically used for modelling tissues where the cells are exposed to two different environments e.g. in the lung, where cells in the air sack (alveolus) are exposed to air from one side and blood from the second side [100]. This microfluidic model is comprised of an upper channel with epithelial and tumor cells in an air flow, an inter-reservoir microporous PDMS membrane, and a lower chamber with epithelial cells, fibroblasts and immune cells in a medium flow mimicking blood circulation (Fig. 3C) [100, 101]. The physiological expiration movements can be simulated by the application of a vacuum in additional side chambers [100]. This method enables the monitoring of cancer cell intravasation into the “blood stream” and cellular crosstalk.

#### Multi-zone multi-layer culture—Cells in Gels in Paper (CiGiP)

This method is based on the use of fabricated chromatography paper sheets with impregnated hydrophobic areas and hydrophilic areas mimicking cell culture plates. The studied cells in liquid ECM hydrogel are pipetted onto hydrophilic areas and left to soak into the paper. The 3D environment is achieved by stacking up to 8 of these cell-paper sheets together to create nutrient and oxygen gradients as in the natural tissue environment (Fig. 3D). The phenotype of the cells growing in layers is similar to the cells growing in the 3D spheroids. The stacking of the layers enables combining various types of cells to be seeded with variable cell density to achieve different ratios of cell types in the experiment as in natural tissue. This can be used to study migration, as cells can freely move to the attractant through paper pores between layers, and the migration of the cells can be traced using microscopy. The thickness of one paper layer is approximately 200 μm, which is thick enough for direct confocal microscopy observation of the seeded cells after the de-stacking of layers [102, 103].

#### Organoids—cultured mini-organs

In general, an organoid is a cellular structure cultured from an induced pluripotent stem cell, which differentiates into organ-specific cell types and forms functional organlike structures [104]. There is a higher order of self-assembly in organoids than in spheroid cultures, and the organoids are more dependent on the ECM during their formation. This approach offers the opportunity to prepare structures created with genetically modified cells using RNAi or CRISPR techniques to model individual processes during tumorigenesis [105].

#### Tumor organoids

Tumor organoids are generated directly from patient-derived tumors and preserve the genetic, histologic, and functional characteristics of the original tumor, including its heterogeneity [104, 106–109] as well as the natural TME [110], which is necessary in cancer models due to the roles of the TME in tumor metastasis, progression and carcinogenesis [111]. First, the tumor is mechanically or enzymatically digested. The obtained mixture including tumor cells, immune cells, vascular cells, fibroblasts etc. and ECM components is combined with a 3D matrix such as collagen or Matrigel® and cultured in GF-reduced medium or in medium supplemented with a specific GF according to the tumor type (Fig. 3E) (reviewed in [104, 112]). The air–liquid interface method combines the growth of cancer and stromal cells into one organoid, which is embedded in acellular collagen in the upper insert of a Boyden chamber (air) with cultivation media containing the GF (liquid) in the bottom well of the Boyden chamber (Fig. 3E) [113]. This approach produces high-fidelity models with multicellular architecture, tissue–tissue interfaces and the physiologically relevant physical microenvironment of cancers growing within living human organs while sustaining vascular perfusion in vitro [75]. Tumor organoid formation is decreased in tumors that underwent tumor-cell-reducing neoadjuvant chemotherapy [104]. The application of a tumor organoid embedded in microfluidic devices makes it possible to modify oxygen pressure, which is fundamental for the tumor cell phenotype during the metastatic process, as a natural TME is hypoxic with an oxygen gradient present [114]. The possibilities of tumor organoids in microfluidic devices facilitate the testing of a number of biological issues (e.g. in the development of personalized anti-cancer therapies [108, 115], modelling the role of specific metastasis-associated genes [116], immunobiology studies [111]; new drug discovery [117] and their toxicology testing [118]). Hybrid organoids, where organoids formed by normal healthy cells are inoculated with tumor cells, were successfully used for modelling the metastatic process in the liver for colorectal carcinoma and for therapy screening [119].

3D techniques can be enriched with various scaffolds to further improve their similarity to natural tissue architecture, as the scaffold itself can promote vascularization and tissue formation [120]. The scaffolds are engineered from natural (e.g. gelatin made from animal collagen; polysaccharides such as alginate, cellulose, chitin, chitosan, hyaluronic acid, agarose, dextran, gellan gum, starch, or chondroitin sulphate [121]) and synthetic (poly(ethylene glycol) (PEG), poly-l-lactic acid (PLLA), polycaprolactone (PCL), and poly(lactic acid-co-caprolactone) (PLACL), poly(lactic-co-glycolic acid) (PLGA) [122]) polymers. The combination of such materials enhances the quality of the 3D environment (e.g. collagen enriched with polysaccharides [123]).

#### Multi-organ microfluidic chip

This method combines different organ-on-chip models together for modelling tumor cell migration from a primary tumor to distant site metastasis, e.g. the migration of tumor cells from the upstream lung “organ” to the downstream “organ” representing the brain, bones or liver [101]. Another study further innovated this approach with the addition of a microporous membrane mimicking the blood–brain barrier between the lung and brain “organs” [124].

## Models for studying metastasis in vivo

In vivo testing is an essential part of research in the field of cancer biology, i.e. studying the molecular basis of the disease, and the development of diagnostic approaches and the development of more effective treatments, including new therapeutics. In contrast to the in vitro experiments described above, mammalian in vivo models make it possible for the experimental conditions to better approximate the complex state of the organism, i.e. conditions in which pathological processes take place which cannot be sufficiently simulated in vitro. Thus, in vivo experiments provide relatively more reliable results, but they are experimentally and economically demanding. The development of genetically modified model organisms has thus become an indispensable branch supporting this research, without which it would not be possible to study physiological and pathological processes in a complex system such as a living organism. A number of organisms have been used to study cell migration and invasiveness in vivo. The amoeba *Dictyostelium discoideum* has fundamentally contributed to the understanding of chemotaxis and the identification of its potential regulators [125]. The roundworm (*Caenorhabditis elegans*), thanks to its transparent body and the possibility of cell visualization using green fluorescent protein (GFP) allows the analysis of natural and pathological migratory pathways [126], as well as the translucent embryos and pupae of the fruit fly (*Drosophila melanogaster*) allow observation of the migration of certain cell types [127, 128]. Fluorescently labelled neutrophils of the zebrafish (*Danio rerio*) were then used, for example, to study cellular processes during inflammation [129, 130].

### Mice—the unrivalled in vivo model in tumor biology

**Fig. 4:**
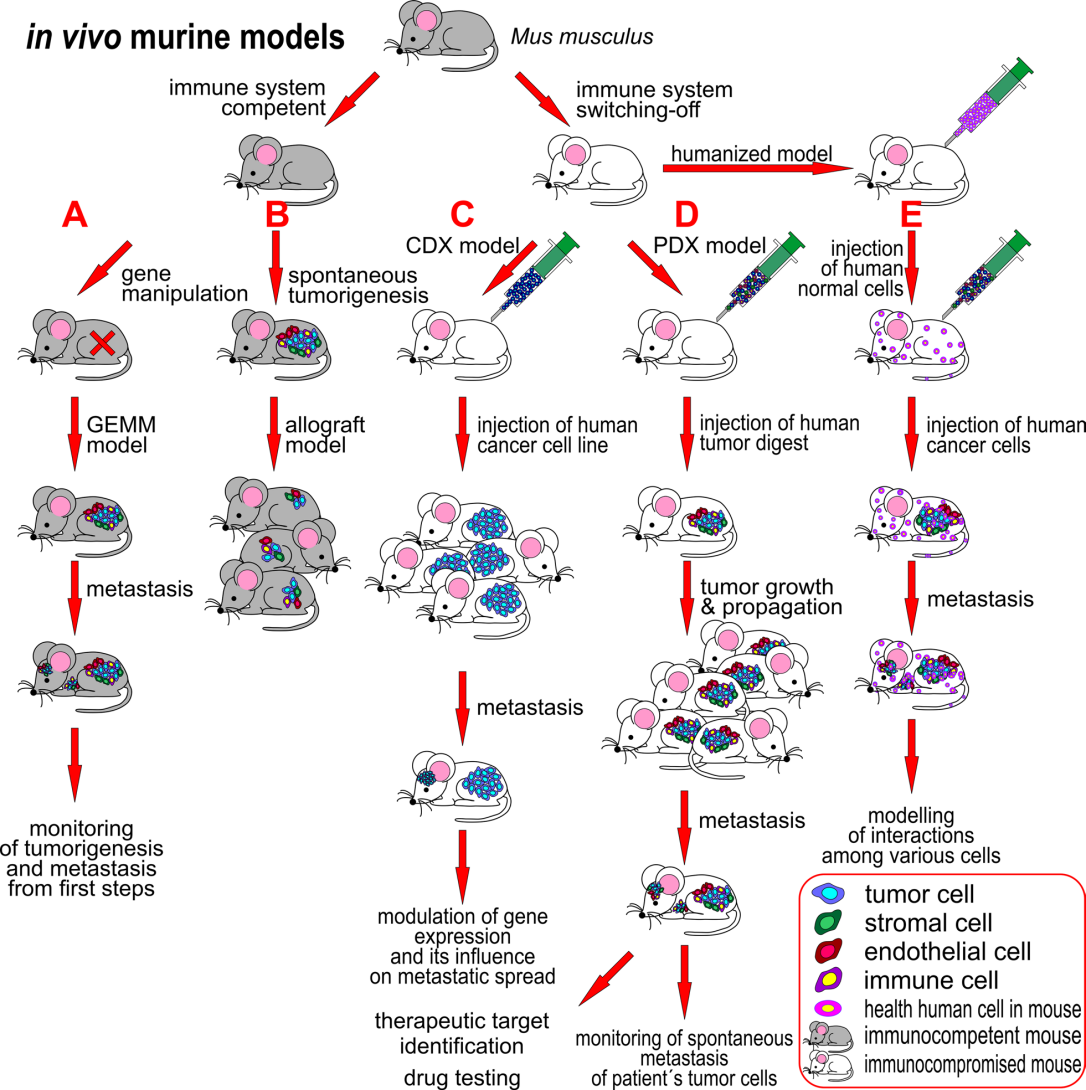
Principles of in vivo studies on mice. *Mus musculus* is the most commonly used animal model in tumor biology, and various strains have been established for research use. Immunocompetent mice (on the left) are used for the cancer research of **A** genetically engineered mouse models (GEMM) and **B** as a syngeneic mouse with spontaneous tumorigenesis. The main advantage of immunocompetent mice is an active immune system, which interacts with and influences the growth of the tumor. Tumorigenesis of the GEMM, where the gene of interest is mutated, can be monitored from the initial steps. Syngeneic mice can be allotransplanted into the same mice strain or the tumor removed to accelerate tumor growth and progression to metastasis. There is a wide range of immunocompromised mice available from non-thymic mice to NOD SCID gamma mice (on the right) for testing various clinical questions. These mice are hosts for the xenotransplantation of human tissues and cells. **C** A cell-line-derived xenograft (CDX) mouse model can be produced easily by the injection of tumor cells from an established tumor cell line carrying the acquired genotype into an appropriate mouse strain. **D** The patient-derived xenograft (PDX) mouse model is produced by the injection of a tumor cell digest into an appropriate mouse strain and through this, a similar environment for tumor cells as in a human tumor is achieved. The tumors can be propagated by the xenotransplantation of growing tumor tissue into the same mouse strain, which significantly shortens the time to tumor cell metastasizing. **E** Humanized mouse models are inoculated with normal human cells (immune, stromal cells) and enable the study of interactions between the tumor and various normal human cells. Models B-E are so-called “transplant models”

However, the most commonly used organism to study the mechanisms of tumor formation, tumor growth and metastatic cascade processes in vivo is the domestic mouse (*Mus musculus*) [131–133]. The study of carcinogenesis in mice enables having a tumor, circulating tumor cells and metastasis in one animal at the same time [134]. Different immune system variants of mice have been established for in vivo research (Fig. 4): (i) immunocompetent mice (capable of an immune response to the antigen), which have been used for preparing the various genetically engineered mice models (GEMMs, [135]), or who have been used as syngeneic mice, that enable the production of allograft mice models [136]; (ii) immunocompromised mice (with the immune response to the antigen decreased to various degrees or completely absent) with deleted components of the specific and innate immune system ranging from athymic nude mouse to NOD SCID gamma (NSG, The Jackson Laboratory) mouse, which enable the engraftment of human material to form xenografts [137], and (iii) humanized mice, which are immunocompromised mice inoculated with human components of the tumor microenvironment such as immune cells, stromal tissue and peripheral blood to better model interactions in human tumors [138–141]. All these models offer the potential to study various aspects and steps in metastasis.

#### Genetically engineered mice models

GEMMs (Fig. 4A) carry essential genetic changes characteristic of the initiation of tumorigenesis in cells interacting with stroma, which enables all metastatic cascade steps to be modelled [135]. The introduced genetic changes include: (i) the insertion of oncogenes under the control of a tissue-specific promoter (e.g. the mammary-specific MMTV promoter for breast tissue), (ii) the knock-out of tumor suppressor genes (e.g. *TP53*), (iii) conditional activation/inactivation expression systems based on the Cre recombinase/*loxP* system enabling the expression of oncogenes and tumor suppressor genes to be switched on or off, (iv) regulatable expression systems based on the Cre-estrogen receptor or tetracycline expression systems that enable the temporal and spatial control of genes driven by estrogen (and blocked by tamoxifen) or doxycycline, respectively, at the tissue, organ or organism level (reviewed in [135]). GEMMs for breast, prostate, lung, pancreatic, colorectal, bladder and ovarian carcinomas have been prepared (reviewed in [142, 143]) to display de novo spontaneous tumor progression and metastasis formation from the initial steps based on genetic changes in the interaction with the immune system and microenvironment in mice [135, 144], but GEMMs are naturally weakly metastatic, and often do not reflect the complex organ tropism of human tumor cells [145]. The low metastatic load can be overcome by removing the primary tumor, after which the subsequent development of metastases can be monitored [146, 147]. Furthermore, the murine organism does not completely fit the human organism (e.g. significantly shorter life span, differences in immune system etc.), so the results may not be generalized at all to human tumorigenesis [135].

### Transplant models

Key considerations in using transplantation models include the choice of host mouse, the mode of inoculation, and selection of the transplantation site, which may have a considerable impact on their relevance for metastasis progression and preclinical investigations [143]. The entry site of tumor cells into the murine organism predicts the locations of metastases: injection into the tail vein leads to lung metastasis, as tumor cells are captured in the first small vasculature they meet in the lungs [148, 149], intracardiac injection spreads cells to the whole body, and distant metastasis in the bones, brain or liver may be observed [150–152], orthotopic injection gives tumor cells a microenvironment similar to their original tissue and this supports the natural spread of cancer cells to distant sites and the formation of metastasis [153], while ectopic (subcutaneous or intravenous) injection often does not result in metastasis growth [143]. Rare delivery injection sites are in a carotid artery (brain metastasis), in the spleen or portal vein (liver metastasis, [154]) or iliac artery (bone metastasis, [155]).

#### Syngeneic mice models

These immunocompetent mice remain the gold standard for studying metastasis [143]. Spontaneous tumor or cancer cells from inbred mouse (e.g. BALB/C, C57BL) are reintroduced to other mice of the same genetic background to produce an allograft mouse model ([136], Fig. 4B). This model was used for the first studies of metastasis in vivo [156]. Nowadays, the most frequent human tumors (e.g. melanoma, breast, colorectal, ovarian, hepatocellular, renal or lung carcinomas) are studied in syngeneic murine models. Although tumors in syngeneic mice are less metastatic, this should be overcome by the implantation of highly metastatic murine cell lines: B16, 4T1, Met-1, RMI and Lewis lung carcinoma cells [135]. The models were used to identify the genes responsible for metastasis regulation in genome-wide studies [157, 158]. The identification of genes suppressing the colonization of the lung by mouse mammary tumor 4T1 cells were performed with 4T1 cells transduced with pools of 48 individual shRNA from an RNAi library (~ 1000 genes with tumor suppressive potential) to express siRNA, which were then intravenously injected into BALB/C mice to grow lung lesions. The next-generation sequencing of DNA isolated from extirpated lung metastases identified individual shRNA and genes involved in metastasis suppression [157]. van der Weyden et al*.* screened the in vivo growth of lung metastasis of B16-F10 murine melanoma cells in syngeneic control and 810 mutant mouse lines. This screen revealed 23 positive or negative microenvironment regulators of lung metastatic colonization according to the number of lung metastatic foci [158]. With breast cancer, orthotopically located tumors in syngeneic mice can be surgically removed and metastasis growth observed over a follow-up period, mimicking the situation in human patients [143, 159].

#### Cell line-derived xenografts and patient-derived xenografts

To better adapt the conditions to humans, xenograft models were established. Producing the xenograft models includes: i) cell line-derived xenograft (CDX; Fig. 4C), in which cultured cells of established tumor cell lines are injected into immunocompromised or humanized mouse, or ii) patient-derived xenograft (PDX; Fig. 4D) mice models, in which the same procedure is done directly with a digested mixture of the patient´s tumor cells or with cut tumor pieces [160–162]. The genetic manipulation of established cell lines makes CDX models usable for testing the role of the modified expression of an individual gene on metastatic process (i.e. metastatic drivers) [163]. As cultured cells in a 2D environment do not retain the characteristics of the original tumor, CDX are consequently poorer models of tumor phenotype than PDX [145], as PDX omits the selective pressures associated with cell culturing and are more likely to reproduce the architecture, heterogeneity, and histopathology of patient tumors than CDX models; nonetheless, their tendency to metastasize is dependent on the parental tumors and immunocompromised mice strain used [143]. The best results in PDX metastatic modeling are achieved by orthotopic transplantation, as is shown in breast cancer models [164]. Utilising a PDX model enables monitoring of the spontaneous metastasis of the patient´s tumor cells as well as studying the effects of various therapeutic strategies for eliminating metastatic spread [165]. The disadvantage of PDX models is their expensive, time-consuming handling and highly precise preparation [107], on the other hand, a large portfolio of PDX models of various tumor types and subtypes is commercially available (e.g. [166, 167]).

#### Humanized patient-derived xenografts

The humanized PDX model (Fig. 4E) is a state-of-the-art solution in in vivo mice models these days: they provide the same level of tumor fidelity and phenotype and give similar results in various applications as tumor organoids [107], enable the monitoring of the emerging metastasis as the inserted tumor cells interact with the surrounding microenvironment, including immune cells, at the given site, and support their metastatic potential and spread according to the entry site into the organism, as orthotopic injection is mostly relevant and increases the incidence of metastasis [168].

#### In vivo imaging

Various in vivo imaging methods, such as magnetic resonance, positron emission tomography, computed tomography, chemiluminescence and fluorescence imaging, and others, are used to macroscopically evaluate the onset and development of metastases. A bioluminiscence detection approach, in which tumor cells carry a luciferase gene and luciferase enzyme activity with a low-molecular substrate and emit visible light to be detected, was implemented as a method for the sensitive and high-throughput evaluation of primary tumor and metastasis growth in a bladder cancer model [165] or in combination with fluorescence in a breast cancer model [169].

## Study of migration and invasiveness in non-solid malignancies

Neoplastic malignancies of hematopoietic or lymphatic tissues including leukemia, lymphoma or myeloma are—due to their natural spread to the whole body in the bloodstream or lymph system—considered to be metastatic at the time of diagnosis [170]. The studies of migrative or invasive properties of leukemic cells are aimed at revealing cytokine-leukemic cell crosstalk and use the Transwell assay principle for this purpose [171–175]. In some cases, leukemia or lymphomas form metastatic lesions in the brain or spinal cord by crossing the blood–brain barrier. An invasive test based on co-cultivation of an endothelial monolayer of non-dividing BMVEC cells with leukemic cells in a Transwell insert filled with ECM mimics the invasion of leukemic cells across the blood–brain barrier [176]. Another migrative test of leukemic cells is based on chemotaxis slide, where the observation chamber is filled with leukemic cells embedded in a 3D ECM hydrogel environment and surrounded by two reservoirs with or without chemoattractant. The migration of leukemic cells toward the chemoattractant gradient is observed using time-lapse microscopy [177]. An in vitro circulatory system (FiberCell System Inc.) can be used for the preparation of a high number of leukemic cells with different phenotypes: capillary-like hollow fibers made of polysulphone are coated with gelatin and colonized with endothelial cells. The circulating leukemic cells freely flow through the hollow fibers, while migratory leukemic cells bind to endothelial cells and migrate to the “extravascular” space [171, 178]. This approach modulates cellular expression as cells react to the hydrodynamic (shear) forces in the circulatory system, and enables the transcriptomic or proteomic analysis of such phenotypically different cells. Another option for studying metastasis in non-solid malignancies are in vivo mice studies: PDX with leukemic cells expressing firefly luciferase and eGFP in NSG mice to see the natural movement of the patient´s cells in the organism [179], CDX using Jurkat cells influenced by various inhibitors and stained with tracker dyes in NSG mice to compare cell migration into the bone marrow and evaluate the role of inhibitors [180].

## Future directions

The use of 3D techniques in cancer research is a leading topic that is providing new data and revealing the unknown consequences. The incorporation of simple as well as complex 3D techniques into routine laboratory practice is proceeding, as two-thirds of researchers perform or plan to perform 3D culture, as HTS technology´s survey showed [181, 182]. This is supported by the growing range of 3D cultureware and protocols for preparing uniform 3D models by biotechnology companies. Tumor organoids, mainly patient-derived tumor organoids thanks to their complexity and similarity to the original tumor, seem to be the best model of tumor phenotype for monitoring invasiveness potential, and testing new anti-cancer therapies, including immunotherapy and the study of drug resistance [182, 183], both with a significant role in personalized medicine and with the possibility of diminishing the use of mice in research. The ongoing development and routine use of microfluidic devices hand in hand with improvements in imaging techniques and bioprinting in combination with the above means that cancer 3D models can provide uniformity and higher complexity, including vasculature, to obtain a high-throughput research system that is able to answer complex clinical questions of metastasis and anti-cancer treatment [184].

In vivo, mainly PDX models made of tumors or patient-derived organoids with humanized components of the tumor microenvironment, especially with a more physiologically relevant immune system, are state-of-the-art techniques whose results may be better transferable to the clinic [134, 143]. There is an increasing trend of combining mice models in one analysis to overcome bias caused by murine types [185]. The important questions of how metastatic cells survive in the premetastatic niche for months or years and what impulse activates dormant cell to grow into a metastasis need to be answered using in vivo mice models [134].

## Conclusion

A great deal of effort by scientists over the last few decades has led to the establishment of a number of 2D/3D in vitro methods and in vivo techniques that are used to study cell migration and invasiveness, especially of cancer cells. These techniques are suitable tools for the research of a wide range of biological processes, with the emphasis on studying the molecular basis of cancer development and processes leading to metastasis. Mainly, 3D techniques are being developed rapidly to diminish the use of in vivo testing in cancer research and overcome its ethical controversy, although it is not possible to completely stop in vivo testing yet. Thus, the large portfolio of various mice models is offered for metastatic research to further improve the findings obtained from 3D models. The application and ongoing development of these techniques provide significant new knowledge leading to a better understanding of the metastatic process, new therapeutic targets, adequate testing of emerging therapeutics and the improvement of personalized cancer treatment.

## Data Availability

Not applicable.
